# Causal relationships between risk of venous thromboembolism and 18 cancers: a bidirectional Mendelian randomization analysis

**DOI:** 10.1093/ije/dyad170

**Published:** 2023-12-20

**Authors:** Naomi Cornish, Philip Haycock, Hermann Brenner, Jane C Figueiredo, Tessel E Galesloot, Robert C Grant, Mattias Johansson, Daniela Mariosa, James McKay, Rish Pai, Andrew J Pellatt, N Jewel Samadder, Jianxin Shi, Florian Thibord, David-Alexandre Trégouët, Catherine Voegele, Chrissie Thirlwell, Andrew Mumford, Ryan Langdon, InterLymph Consortium, INVENT-MVP Consortium

**Affiliations:** School of Cellular and Molecular Medicine, University of Bristol, Bristol, UK; University of Exeter Medical School, University of Exeter, Exeter, UK; Medical Research Council Integrative Epidemiology Unit, University of Bristol, Bristol, UK; Division of Clinical Epidemiology and Aging Research, German Cancer Research Center (DKFZ), Heidelberg, Germany; Division of Preventive Oncology, German Cancer Research Center (DKFZ) and National Center for Tumor Diseases (NCT), Heidelberg, Germany; German Cancer Consortium (DKTK), German Cancer Research Center (DKFZ), Heidelberg, Germany; Department of Medicine, Samuel Oschin Comprehensive Cancer Institute, Cedars-Sinai Medical Center, Los Angeles, CA, USA; Department for Health Evidence, Radboud University Medical Center, Nijmegen, The Netherlands; Division of Medical Oncology and Hematology, Princess Margaret Cancer Centre, University Health Network, Toronto, ON, Canada; International Agency for Research on Cancer, World Health Organization, Lyon, France; International Agency for Research on Cancer, World Health Organization, Lyon, France; International Agency for Research on Cancer, World Health Organization, Lyon, France; Department of Laboratory Medicine and Pathology, Mayo Clinic, Scottsdale, AZ, USA; Division of Cancer Medicine, MD Anderson Cancer Center, Houston, TX, USA; Division of Gastroenterology, Mayo Clinic, Phoenix, AZ, USA; Division of Cancer Epidemiology and Genetics, National Cancer Institute, National Institutes of Health, Bethesda, MD, USA; Population Sciences Branch, Division of Intramural Research, National Heart, Lung and Blood Institute, Framingham, MA, USA; University of Bordeaux, Bordeaux Population Health Research Center, Bordeaux, France; International Agency for Research on Cancer, World Health Organization, Lyon, France; University of Exeter Medical School, University of Exeter, Exeter, UK; Bristol Medical School, University of Bristol, Bristol, UK; School of Cellular and Molecular Medicine, University of Bristol, Bristol, UK; Medical Research Council Integrative Epidemiology Unit, University of Bristol, Bristol, UK

**Keywords:** Mendelian randomization, genetic epidemiology, deep vein thrombosis, pulmonary embolus, malignancy

## Abstract

**Background:**

People with cancer experience high rates of venous thromboembolism (VTE). Risk of subsequent cancer is also increased in people experiencing their first VTE. The causal mechanisms underlying this association are not completely understood, and it is unknown whether VTE is itself a risk factor for cancer.

**Methods:**

We used data from large genome-wide association study meta-analyses to perform bidirectional Mendelian randomization analyses to estimate causal associations between genetic liability to VTE and risk of 18 different cancers.

**Results:**

We found no conclusive evidence that genetic liability to VTE was causally associated with an increased incidence of cancer, or vice versa. We observed an association between liability to VTE and pancreatic cancer risk [odds ratio for pancreatic cancer: 1.23 (95% confidence interval: 1.08–1.40) per log-odds increase in VTE risk, *P = *0.002]. However, sensitivity analyses revealed this association was predominantly driven by a variant proxying non-O blood group, with inadequate evidence to suggest a causal relationship.

**Conclusions:**

These findings do not support the hypothesis that genetic liability to VTE is a cause of cancer. Existing observational epidemiological associations between VTE and cancer are therefore more likely to be driven by pathophysiological changes which occur in the setting of active cancer and anti-cancer treatments. Further work is required to explore and synthesize evidence for these mechanisms.

Key Messages

- There is strong observational evidence that active cancer is associated with venous thromboembolism.
- It is currently unknown whether venous thromboembolism is a risk factor for cancer.
- We applied a bidirectional Mendelian randomization framework to appraise the causal relationships between genetic liability to venous thromboembolism and 18 different cancers.
- Overall, there was no clear evidence from Mendelian randomization that lifetime-elevated risk of venous thromboembolism is causally associated with an increased risk of cancer, or visa versa.

## Introduction

Venous thromboembolism (VTE), which includes deep vein thrombosis and pulmonary embolism, is the third most common cause of death from cardiovascular disease globally.^1^ Over 20% of all VTE events occur in people with pre-existing cancer, for whom the relative risk of VTE is at least five times higher than age-matched non-cancer controls.^2^ Evidence from in-vitro and animal models shows that many tumours directly activate platelets, produce procoagulant proteins or alter the vascular endothelium, all of which may increase the risk of thrombosis.^3^ Systemic anti-cancer therapy or surgery and their resultant complications, including sepsis and hospitalization, are also powerful risk factors for VTE.^2^

It is currently unknown whether elevated VTE risk is causally associated with cancer incidence. Over 5% of people presenting with a first VTE are subsequently diagnosed with cancer within the ensuing year,^4^^,^^5^ and several studies have indicated that cancer risk may be elevated over the longer term for people with a history of VTE.^6–9^ Experiments in mice indicate that pro-thrombotic proteins, including tissue factor and fibrinogen, facilitate tumour growth, survival and metastasis.^10^^,^^11^ However, observational studies examining whether treatment with antiplatelet or anticoagulant medication reduces risks of cancer have shown conflicting results.^12–14^

Attempts to elucidate complex causal relationships between VTE and cancer using traditional observational studies are complicated by difficulties in ascertaining direction of causality, and are susceptible to unmeasured and residual confounding from risk factors which are common to both VTE and cancer, including smoking, obesity and co-existing inflammatory conditions.^2^ Mendelian randomization (MR) addresses some of these limitations. It employs genetic variants, typically single nucleotide polymorphisms (SNPs), as instrumental variables (IVs) to proxy the effect of an exposure on an outcome. As SNPs are randomly allocated and fixed at conception, they are unconfounded by acquired and environmental risk factors.^15^

Recently Chen *et al.* used MR to explore the unidirectional causal effect of liability to 14 cancers on VTE risk, and reported that genetically elevated risks of breast cancer and lymphoma may be causally associated with VTE.^16^ However, this study was limited by relatively small sample sizes (5403 VTE cases and a median of 2321 cancer cases). Here, we use genome-wide association study (GWAS) meta-analysis data, derived from large cancer-specific consortia and >70 000 VTE cases, to perform bidirectional MR analyses of the effect of genetic liability to VTE on the risk of 18 cancers, and conversely the effect of genetic liability to cancer on the risk of VTE.

## Methods

### Data sources and genetic instruments

We obtained European-ancestry summary genetic data from GWAS meta-analyses examining risk of VTE and 18 common cancers, respectively (Table 1). Case definitions and covariate adjustment for each GWAS are shown in Supplementary Table S1 (available as Supplementary data at *IJE* online).

**Table 1.** Source of genome-wide association study data used for bidirectional Mendelian randomization analyses

Trait	Study, date	No. of cases	No. of controls	Sample size
Venous thromboembolism	Thibord *et al.*, 2022^17^	71 771	1 059 740	1 131 511
Breast cancer	Zhang *et al.*, 2020^18^	133 384	113 789	247 173
Prostate cancer	Schumacher *et al.*, 2018^19^	79 194	61 112	140 306
Endometrial cancer	O’Mara *et al.*, 2018^20^	12 906	108 979	121 885
Colorectal cancer	Huyghe *et al.*, 2019^21^	55 168	65 160	120 328
Melanoma	Landi *et al.,* 2020^22^	30 134	81 415	111 549
Lung cancer	McKay *et al.*, 2017^23^	29 266	56 450	85 716
Ovarian cancer	Phelan *et al.*, 2017^24^	25 509	40 941	66 450
Kidney cancer	Scelo *et al.*, 2017^25^	10 784	20 406	31 190
Oesophageal cancer	Gharahkhani *et al.,* 2016^26^	4112	17 159	21 271
Pancreatic cancer	PanScan/PanC4, 2022^a^	9055	7203	16 258
Diffuse large B cell lymphoma	Cerhan *et al.,* 2014^27^	3857	7666	11 523
Chronic lymphocytic leukaemia	Berndt *et al.,* 2013^28^	3100	7667	10 767
Follicular lymphoma	Skibola *et al.*, 2014^29^	2728	7758	10 468
Oral cancer	Lesseur *et al.*, 2016^30^	2700	5984	8684
Oropharyngeal cancer	Lesseur *et al.*, 2016^30^	2433	5984	8417
Glioma	Melin *et al.*, 2017^31^	4572	3286	7858
Marginal zone lymphoma	Vijai *et al.*, 2015^32^	825	6221	7046
Bladder cancer	Nijmegen Bladder Cancer Study^a^	1799	4745	6544

a Indicates unpublished data.

To examine the association between genetic liability to VTE and each cancer, we extracted risk SNPs associated with VTE at *P *<5 x 10^−8^ from a VTE GWAS conducted by Thibord *et al*.^17^ We clumped SNPs to ensure independence,^33^^,^^34^ then extracted summary statistics for these SNPs from each cancer risk GWAS. We harmonized exposure and outcome data to ensure that effect estimates corresponded to the same allele for each SNP. Full details of the clumping and harmonization process are described in Supplementary Methods (available as Supplementary data at *IJE* online).

To perform the analysis in the opposite direction (with genetic liability to cancer as an exposure and VTE as an outcome), we used the same process and thresholds described above to select independent risk SNPs for each cancer from the relevant cancer GWAS (at *P *<5 x 10^−8^), then looked up summary statistics for the cancer risk SNPs in the VTE GWAS.

### Statistical analyses

Causal estimates from MR are underpinned by three core assumptions: (i) the genetic variants used as IVs are strongly associated with the exposure; (ii) there are no confounders of the genotype-outcome relationship; (iii) the genetic variants affect the outcome only via the exposure and not through an alternative pathway.^35^

We estimated the r^2^ (variance in phenotype explained by each IV) as described by Lee *et al.,*^36^ using an assumed VTE prevalence of 0.2%.^17^ Prevalence estimates for each cancer were obtained from the International Agency for Research on Cancer.^37^ We assessed the strength of each SNP-exposure association using F statistics, estimated from summary data by dividing the square of the effect size (log-odds) by the square of the standard error. We performed Steiger filtering to exclude SNPs which explained more variance (r^2^) in the outcome than the exposure.^38^ Assuming a causal relationship exists between an exposure and an outcome, a robust genetic IV should only explain the proportion of variance in the outcome which relates to the effect of the exposure.^15^ Steiger filtering identifies SNPs which have a disproportionately large effect on the outcome compared with the exposure, reducing the risk of using invalid IVs which impact on the outcome via horizontal pleiotropy or which proxy a reverse causal pathway from outcome to exposure.

As recommended by published guidelines,^35^ we used an inverse variance-weighted multiplicative random effects MR model (MR-IVW) for the primary analysis, with correction for under-dispersion when only a few SNPs were available for analysis. The MR-IVW is derived from a linear regression of the SNP-outcome and SNP-exposure associations, with each SNP weighted according to the inverse of the variance of the SNP-outcome effect. We assessed heterogeneity between the individual SNP estimates in the MR-IVW using Cochran’s Q statistic. The exception to this was for marginal zone lymphoma, where only a single variant was available as a proxy; therefore the Wald ratio estimator^15^ was used to estimate the causal effect.

Since MR-IVW assumes there is no directional pleiotropy in the MR instruments, we performed a range of sensitivity analyses, including MR-Egger, weighted-median, weighted-mode and leave-one-out analyses to test this assumption.^39^ For phenotypes with at least three SNPs available as a proxy, we also used the MR PRESSO test to evaluate for horizontal pleiotropy and remove outlying SNPs.^40^

The VTE GWAS data came from a discovery cohort where some novel variants had not been replicated. Therefore, to evaluate for bias resulting from either weak instruments or ‘the winner’s curse’,^41^ we performed a sensitivity analysis where we limited genetic instruments for VTE to replicated loci only.^17^

Previous studies have reported that two powerful VTE risk variants, Factor V Leiden (rs6025, A allele) and Prothrombin G20210A (rs1799963, A allele), may be associated with cancer incidence.^42–44^ These variants have a prevalence of ∼5% and ∼1%, respectively, in European populations. Notably, carriers of either of these variants have a VTE risk which is 3–5 times higher than those with wild-type alleles.^45^ The Prothrombin G20210A variant was excluded from the main VTE IV during the clumping process (due to absence from the reference panel). However, we performed a secondary analysis using MR Wald ratios^15^ to examine the association between VTE risk, as proxied by these individual SNPs and risk of each cancer.

Results are presented in accordance with STROBE-MR guidelines^46^ as the odds ratio (OR) and 95% confidence interval (CI) for each outcome per log-odds increase in the risk of exposure. *P*-values (*P*) have been adjusted for multiple testing using a false-discovery rate correction (*FDR-P*). All analyses were performed in R version 4.0.3 using the ‘TwoSampleMR’^33^ and MR-PRESSO^40^ packages.

## Results

### Mendelian randomization analyses of the association between genetic liability to venous thromboembolism and cancer

After selecting independent VTE-risk SNPs (*P *<5 x 10^−8^, r^2^≤0.001), there were 73 SNPs available as genetic instruments for VTE. These variants explained approximately 3% of the variance in VTE risk in the VTE GWAS cohort.^17^^,^^33^^,^^36^ The number of instrumental variables varied for each VTE-cancer analysis (Table 2), as some VTE SNPs were either unavailable for assessment in the cancer GWAS studies, were excluded by Steiger filtering or could not be harmonized between the datasets due to coding-strand ambiguities. Summary data for the SNPs used in each analysis are shown in Supplementary Table S2 (available as Supplementary data at *IJE* online).

**Table 2.** Number of genetic instruments for venous thromboembolism used for Mendelian randomization analyses, associated r^2^ and mean F statistic

Outcome GWAS	**VTE SNPs available** ^a^	**SNPs excluded** ^a^	**VTE SNPs used** ^a^	r^2^ for VTE	Mean F statistic
Breast cancer	68	4	64	0.031	208
Prostate cancer	66	2	64	0.031	210
Endometrial cancer	73	7	66	0.032	205
Colorectal cancer	68	2	66	0.031	204
Melanoma	68	4	64	0.031	209
Lung cancer	64	4	60	0.031	219
Ovarian cancer	68	6	62	0.031	213
Kidney cancer	70	9	61	0.031	218
Oesophageal cancer	70	6	64	0.032	210
Pancreatic cancer	45	5	40	0.023	254
Diffuse large B cell lymphoma	71	16	55	0.030	237
Chronic lymphocytic leukaemia	71	23	48	0.030	266
Follicular lymphoma	71	23	48	0.029	264
Oral cancer	68	15	53	0.030	242
Oropharyngeal cancer	68	18	50	0.029	255
Glioma	69	26	43	0.028	283
Marginal zone lymphoma	71	29	42	0.028	292
Bladder cancer	66	26	40	0.028	307

GWAS, genome-wide association study; r^2^, phenotypic variance explained; SNP, single nucleotide polymorphism; VTE, venous thromboembolism.

a For each cancer outcome GWAS, ‘VTE SNPs available’ refers to the number of VTE-risk SNPs for which a direct correlate or proxy could be identified in the cancer GWAS study; ‘SNPs excluded’ refers to the number of VTE SNPs which could not be harmonized due to coding-strand ambiguities or which were excluded after Steiger filtering. ‘VTE SNPs used’ refers to the final number of genetic instruments for VTE used in each analysis.

**Figure 1.**
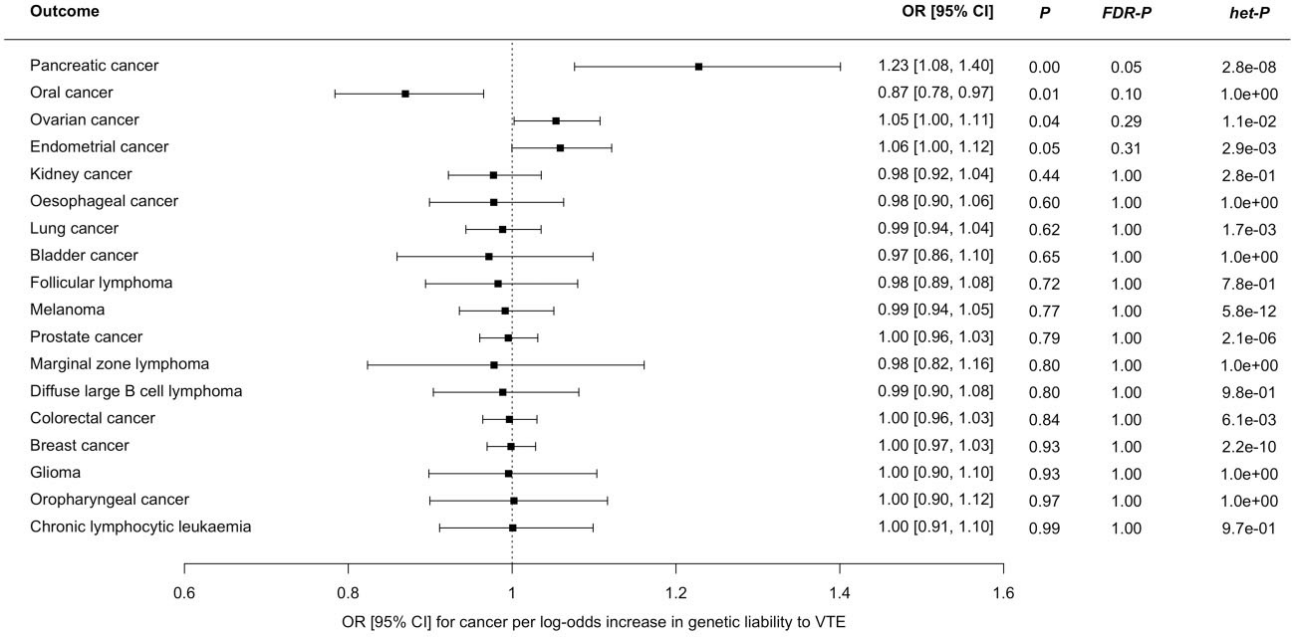
Forest plot showing estimates from Mendelian randomization inverse variance-weighted estimates of the effect of genetic liability to venous thromboembolism as an exposure on 18 cancers as outcomes. CI, confidence interval; FDR-P, false-discovery corrected *P-*value; het-P, heterogeneity *P-*value for Cochran’s Q statistic; OR, odds ratio; VTE, venous thromboembolism

**Figure 2.**
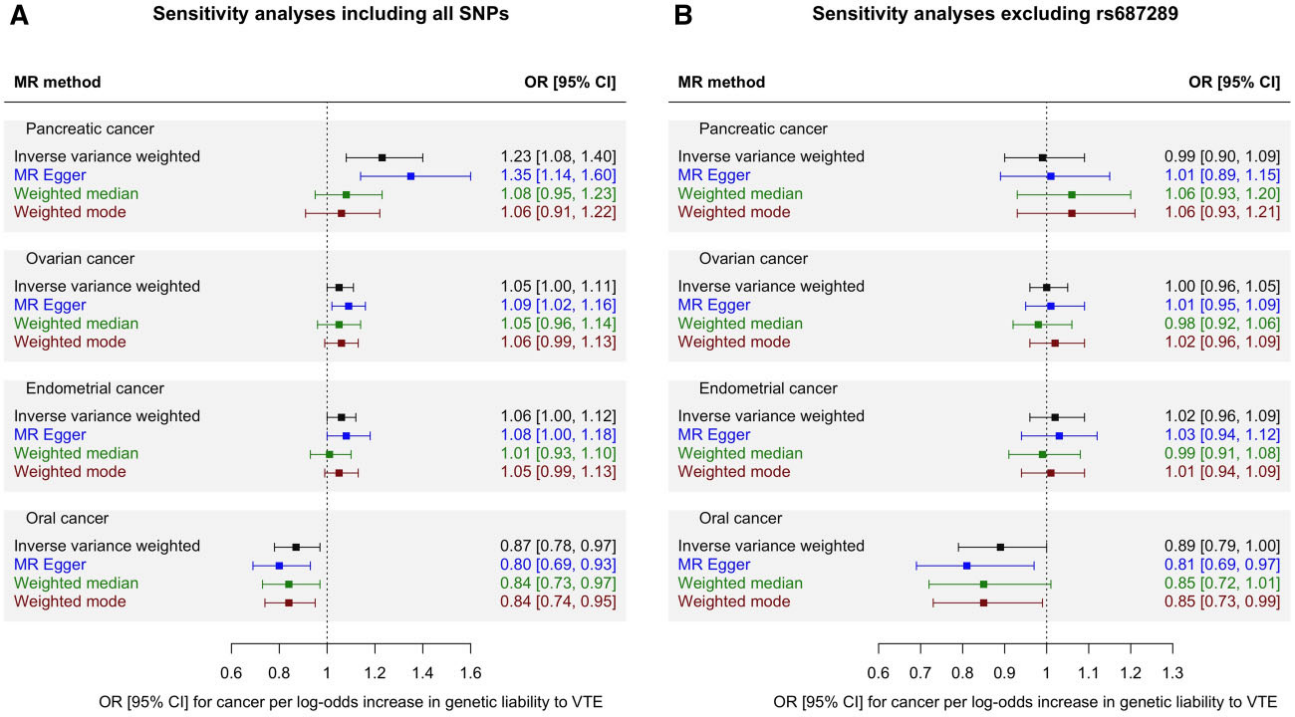
Mendelian randomization sensitivity analyses of genetic liability to venous thromboembolism as an exposure and risk of four cancers (pancreatic, ovarian, endometrial and oral cancer) which showed an association (*P* ⩽0.05) in the MR-IVW analysis. (A) shows sensitivity analyses including all SNPs. (B) shows sensitivity analyses with rs687289 removed. CI, confidence interval; FDR-P, false-discovery corrected *P-*value; het-P, heterogeneity *P-*value for Cochran’s Q statistic; MR-IVW, Mendelian randomization inverse variance-weighted estimates; OR, odds ratio; SNP, singlenucleotide polymorphism; VTE, venous thromboembolism

We estimated the OR for each cancer per log-odds increase in genetic liability to VTE using MR-IVW analysis (Figure 1). Increased risk of VTE was associated with an increased risk of pancreatic cancer [OR 1.23 (95% CI, 1.08–1.40), *P *=* *0.002, *FDR-P = *0.05]. A much weaker association in the same direction was seen for ovarian cancer (OR 1.05 (95% CI, 1.00–1.11), *P *=* *0.04, *FDR-P = *0.29] and endometrial cancer [OR 1.06 (95% CI, 1.00–1.12), *P *=* *0.05, *FDR-P = *0.31]. Sensitivity analyses showed inconsistent estimates of effect between the MR-IVW, MR-Egger, weighted-median and weighted-mode estimates for pancreatic cancer (Figure 2A). There was significant heterogeneity in the VTE IV estimates for pancreatic, ovarian and endometrial cancer as assessed by Cochran’s Q statistic, and the MR-PRESSO global test showed evidence for horizontal pleiotropy (Supplementary Table S3, available as Supplementary data at *IJE* online). Graphical assessment of the leave-one-out plots, single SNP plots and funnel plots identified an outlying SNP (rs687289) which was confirmed by the MR-PRESSO outlier test (Supplementary Figures S1–S3, available as Supplementary data at *IJE* online). Removal of this SNP from the analysis virtually abolished the association between VTE and pancreatic, ovarian and endometrial cancer (Figure 2B).

There was weak evidence from the MR-IVW analysis for a small inverse association between genetic risk of VTE and risk of oral cancer [OR 0.87 (95% CI, 0.78–0.97), *P *=* *0.01, *FDR-P = *0.10]. Sensitivity analyses showed consistent estimates of effect (Figure 2) with no indication of directional pleiotropy identified from the MR-Egger or MR-PRESSO analysis (Supplementary Table S3, available as Supplementary data at *IJE* online). There were no obvious outliers on inspection of the funnel plots and leave-one-out plots (Supplementary Figures S1 and S2, available as Supplementary data at *IJE* online).

We performed two additional sensitivity analyses: first using only 31 replicated VTE SNPs as instrumental variables; secondly using all available VTE SNPs with no Steiger filtering applied. These results were similar to the primary analysis for all 18 cancers (Supplementary Tables S4 and S5, Supplementary Figures S4 and S5, available as Supplementary data at *IJE* online).

**Figure 3.**
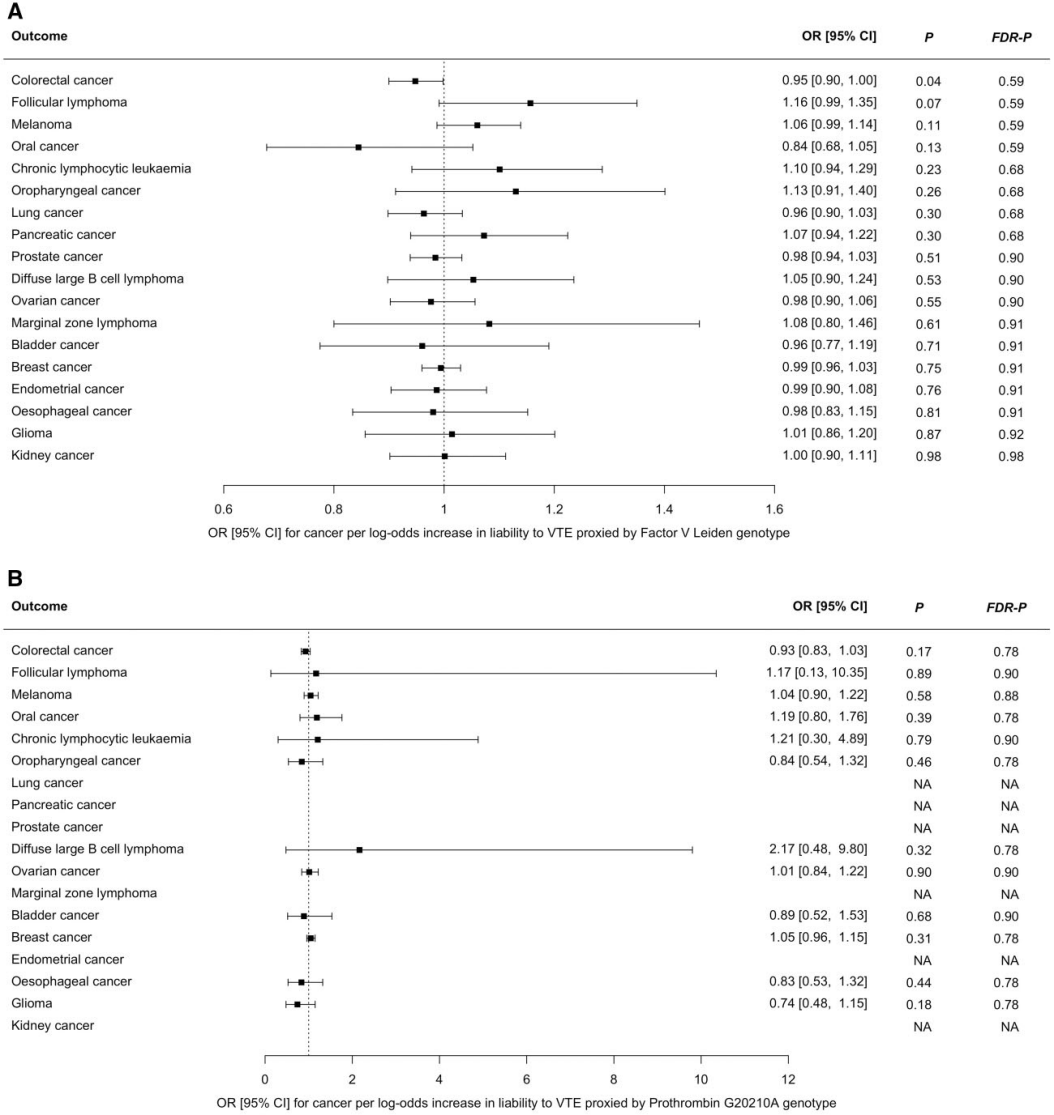
Mendelian randomization Wald ratios for association between (A) liability to VTE, proxied by Factor V Leiden only, and risk of 18 cancers; and (B) liability to VTE, proxied by Prothrombin G20210A only, and risk of 12 cancers. NA indicates cancers for which the Prothrombin G20210A variant was not available in the genome-wide association study summary data. CI, confidence interval; FDR-P, false-discovery corrected *P-*value; OR, odds ratio; VTE, venous thromboembolism

We examined the MR Wald ratios for the association between VTE risk proxied by either Factor V Leiden (rs6025) or Prothrombin G20210A (rs1799963), and cancer (Figure 3; Supplementary Table S6, available as Supplementary data at *IJE* online). Summary data for Factor V Leiden were available for all cancers. Summary data for Prothrombin G20210A were unavailable for six of 18 cancers (endometrial cancer, kidney cancer, lung cancer, marginal zone lymphoma, pancreatic cancer and prostate cancer). There was a very weak inverse association between VTE risk, proxied by Factor V Leiden, and colorectal cancer [OR 0.95 (95% CI, 0.90–1.00), *P = *0.04, *FDR-P *=* *0.59]. There were no associations between Factor V Leiden or Prothrombin G20210A and any other cancer.

### Mendelian randomization analyses of the association between genetic liability to cancer and venous thromboembolism

We performed the MR analyses in the reverse direction, using genetic liability to cancer as an exposure and VTE as an outcome. The number of instrumental variables used for each cancer is shown in Table 3. Summary data for the SNPs used in each analysis are shown in Supplementary Table S7 (available as Supplementary data at *IJE* online).

**Table 3.** Number of genetic instruments for cancer used for Mendelian randomization analyses, associated r^2^ and mean F statistic

Exposure GWAS	**Cancer-risk SNPs** ^a^	**SNPs unavailable** ^a^	**SNPs excluded** ^a^	**Cancer SNPs used** ^a^	r^2^ for cancer	Mean F statistic
Breast cancer	156	1	5	150	0.037	90
Prostate cancer	137	6	8	123	0.063	108
Endometrial cancer	16	0	0	16	0.013	44
Colorectal cancer	56	0	3	53	0.021	64
Melanoma	38	0	1	37	0.052	211
Lung cancer	15	1	0	14	0.013	83
Ovarian cancer	12	0	1	11	0.010	71
Kidney cancer	18	0	0	18	0.034	70
Oesophageal cancer	5	0	0	5	0.011	32
Pancreatic cancer	9	0	1	8	0.015	54
Diffuse large B cell lymphoma	4	0	0	4	0.012	27
Chronic lymphocytic leukaemia	8	0	0	8	0.048	70
Follicular lymphoma	2	0	0	2	0.032	48
Oral cancer	6	0	0	6	0.018	35
Oropharyngeal cancer	3	0	0	3	0.014	38
Glioma	5	0	0	5	0.028	80
Marginal zone lymphoma	1	0	0	1	0.009	10
Bladder cancer	2	0	0	2	0.006	31

GWAS, genome-wide association study; IV, instrumental variable; r^2^, phenotypic variance explained; SNP, single-nucleotide polymorphism; VTE, venous thromboembolism.

a For each cancer exposure GWAS, ‘Cancer-risk SNPs’ refers to the total number of SNPs which could potentially be used as an IV for each cancer; ‘SNPs unavailable’ refers to the number of cancer SNPs for which no direct correlate could be identified in the VTE GWAS study; ‘SNPs excluded’ refers to the number of SNPs which could not be harmonized due to coding-strand ambiguities or which were excluded after Steiger filtering. ‘Cancer SNPs used’ refers to the final number of genetic instruments for cancer used in each analysis.

**Figure 4.**
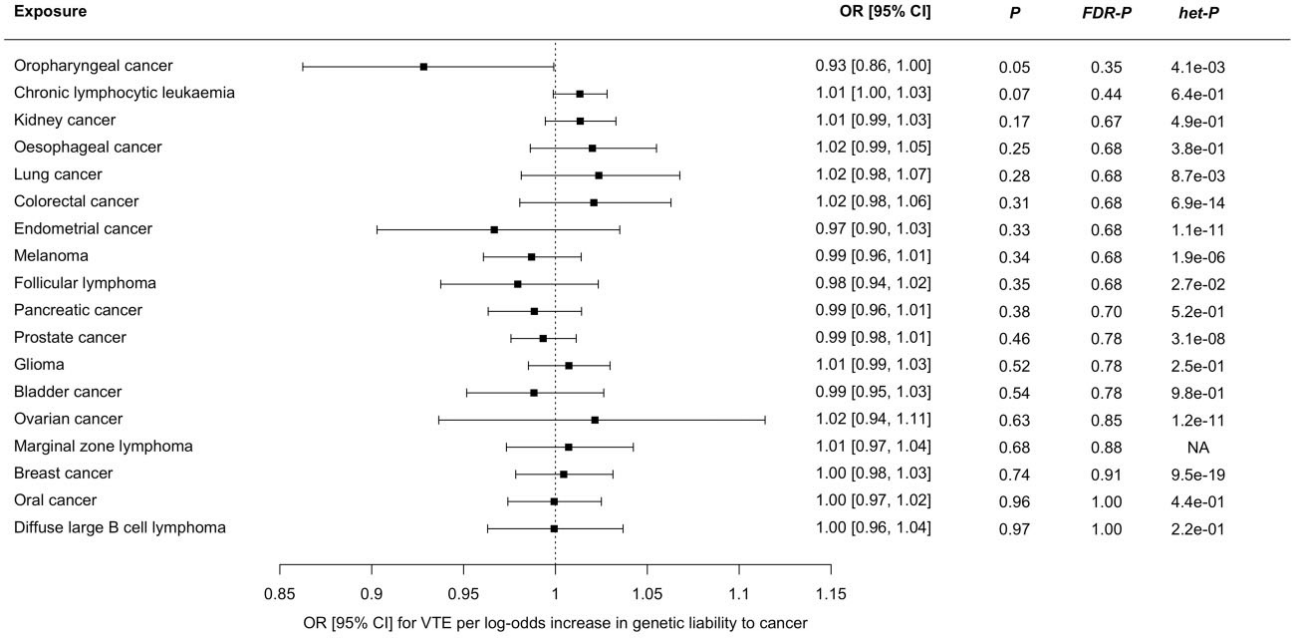
Forest plot showing estimates from Mendelian randomization analyses of the effect of genetic liability to 18 cancers as exposures on risk of venous thromboembolism as an outcome. The Mendelian randomization inverse variance-weighted estimates are shown for all cancers except marginal zone lymphoma, where the Wald ratio is shown, as only a single instrumental variable was available. CI, confidence interval; FDR-P, false-discovery corrected *P-*value; het-P, heterogeneity *P-*value for Cochran’s Q statistic; OR, odds ratio; VTE, venous thromboembolism

The MR-IVW analysis showed very weak evidence for a small inverse association between genetic liability to oropharyngeal cancer and risk of VTE [OR 0.93 (95% CI, 0.86–1.00), *P *=* *0.05, *FDR-P = *0.48; Figure 4]. Only three SNPs were available as instrumental variables, which precluded MR-PRESSO analysis. However, these SNPs displayed significant heterogeneity as measured by Cochran’s Q statistic (*P = *0.004). This was reflected by the wide estimate of effect in the MR Egger analysis [OR 0.97 (95% CI, 0.61–1.52), *P = *0.90, MR Egger intercept −0.02, intercept standard error 0.08]. Full results are shown in Supplementary Table S8 (available as Supplementary data at *IJE* online). There were no other associations between genetically proxied risk of cancer and VTE in the primary analysis.

## Discussion

We observed an association between genetic liability to VTE and increased risk of pancreatic cancer. Pancreatic cancer has consistently been linked with very high rates of VTE in conventional epidemiological studies.^47–49^ However, our MR sensitivity analyses indicated that the association between genetic liability to VTE and pancreatic cancer was largely driven by a single outlying SNP (rs687289). We observed weaker associations between genetic liability to VTE and risks of ovarian and endometrial cancer. Evidence for these associations was minimal after correction for multiple testing, and attenuated further towards the null in sensitivity analyses with rs687289 removed.

The variant rs687289 is found in intron 2 of the *ABO* blood group gene. The VTE-risk allele at this SNP is in high linkage disequilibrium with an allele which determines non-O blood group (rs8176719).^50^ Non-O blood group is associated with increased risk of a range of phenotypes including cardiovascular disease and several cancers, including pancreatic and ovarian cancer.^51^^,^^52^ One possibility is that the MR association between VTE and pancreatic cancer results from horizontal pleiotropy (i.e. rs687289 exerting an effect on both VTE and pancreatic cancer through independent biological pathways). People with non-O blood group have higher levels of von-Willebrand factor and LDL-cholesterol, both of which may potentially contribute to VTE.^50^ The mechanism by which blood group affects cancer risk is unknown, although it is hypothesized that ABO antibodies interact with aberrant glycoproteins expressed on pancreatic tumour cells.^53^ It is also plausible that the association between VTE and pancreatic cancer is mediated by ABO blood group. Further multivariable MR analyses were beyond the scope of this study but would be helpful in evaluating this hypothesis. Last, since the prevalence of different ABO blood groups varies geographically,^54^ the associations driven by this SNP could indicate confounding by population stratification. Although all GWAS data were drawn from genetically inferred European-ancestry participants, this encompasses a heterogeneous population, defined by different GWAS using varying principal component clustering models. Therefore, there may be genetic drift between the cohorts included in the VTE and cancer studies.

There was weak evidence from both the MR-IVW and MR sensitivity analyses that genetic liability to VTE was associated with slightly reduced risk of oral cancer [MR-IVW OR 0.87 (95% CI, 0.78–0.97), *P *=* *0.01, *FDR*-*P = *0.10]. This is surprising, given that procoagulant proteins are frequently over-expressed by oral cancer cells, which suggests growth of this tumour occurs in a prothrombotic environment.^55^ Paradoxically, epidemiological studies have shown that for people presenting with VTE, the risk of subsequent oral cancer diagnosis is relatively low compared with other cancers,^56^ an observation which adds credibility to our results. However, since most people who experience VTE start treatment with anticoagulant therapy,^4^ this could theoretically confound any associations between VTE and oral cancer.^12^ A few small studies have previously described that SNPs in genes encoding two coagulation proteins, plasminogen activator inhibitor 1 (PAI-1) (rs1799889) and Factor XIII (rs5985), respectively, are associated with risk of oral squamous cell carcinoma.^57^^,^^58^ Neither of these SNPs were identified as VTE-risk variants in the VTE GWAS,^17^ and therefore these were not represented in our analysis. Their specific role in oral cancer carcinogenesis is unclear. Given the lack of a consistent biological mechanism to explain our MR finding, our result should be interpreted with caution.

For the MR analyses in the cancer-VTE direction, which examined genetic liability to 18 cancers as exposures and VTE as an outcome, we found no clear evidence that genetic predisposition for any cancer was associated with increased risk of VTE, after correction for multiple testing. There are several caveats which should be considered in the interpretation of this result. This analysis estimates the impact of lifetime elevated genetic risk of cancer on risk of VTE, and will not capture time-dependent causal associations which occur due to acute changes in the context of active or progressive malignancy. Biological mechanisms leading to VTE include interruption to venous blood flow, vascular endothelial dysfunction and/or blood hypercoagulability.^3^ The pathophysiology of VTE in people with cancer may be different from that in people who develop unprovoked VTE (in the absence of cancer): for example, tumour-induced endothelial hypoxia may contribute to VTE in the former group but not the latter.^3^ As our outcome VTE GWAS cohort was derived from a heterogeneous case group (rather than a cancer-specific cohort), this may have reduced our power to detect causal associations between genetic liability to cancer and VTE.^17^ Future MR studies, using IVs which proxy time-dependent or intermediate exposure phenotypes, may be helpful to explore the association between cancer and VTE.

### Study limitations

In addition to the aforementioned limitations, we acknowledge that power to detect causal associations between cancer and VTE may have been limited for cancers with smaller GWAS case numbers. Since VTE in the context of cancer has a high mortality rate,^2^ it is also likely that cancer patients are under-represented in the VTE GWAS meta-analysis due to survival bias and selection bias of some of the contributing sub-studies.^17^ This may distort the ability to detect causal associations with an MR approach.

Given the long latency period of many cancers^41^ and polygenic nature of both cancer and VTE, the possibility of mis-attributing the direction of causality remains a concern. To address this, we performed a bidirectional analysis and used Steiger filtering^38^ to select instrumental variables for each analysis (Supplementary Tables S2 and S7). Interestingly, in the MR of liability to cancer (exposure) on VTE (outcome), this process excluded a single SNP in the *ABO* blood group gene from the IV of both pancreatic and ovarian cancer (rs687289 and rs115478735, respectively). Steiger filtering may be unreliable if there is a significant difference in measurement error between the exposure and outcome GWAS.^38^ We performed a sensitivity analysis in which no Steiger filtering was used, and found broadly similar results to the primary analysis (Supplementary Figure S6) but with much wider estimates for the effect of liability to pancreatic and ovarian cancer on VTE. This may be worth re-exploring in future when larger GWAS for these cancers become available.

### Comparison with wider literature

Although associations between VTE and cancer have been rigorously investigated with conventional epidemiological approaches,^2^ to our knowledge there are no published MR analyses dedicated to examining the causal effect of genetic liability to VTE on cancer risk. One previous MR study has examined the causal effect of genetic liability to multiple cancers on VTE risk.^16^ The authors of this study reported a trend towards reduced VTE risk in the context of genetic predisposition to melanoma, and increased VTE risk in the context of genetic predisposition to non-Hodgkin lymphoma and breast cancer, although evidence for these associations diminished after correction for multiple testing. In contrast our study, which used data from GWAS with much larger case numbers for both VTE and each cancer, did not replicate these associations. Qin *et al.* have also recently reported a unidirectional MR analysis examining the effect of genetic liability to breast cancer on VTE risk, and similarly did not find any association.^59^

Several small case-control studies have applied regression analyses to examine whether carriers of single thrombophilia gene polymorphisms, including Factor V Leiden (rs6025) and Prothrombin G20210A (rs1799963), are at increased risk of cancer.^60^ Two groups previously reported that Prothrombin G20210A was associated with an increased risk of gastrointestinal and colorectal cancer, respectively.^42^^,^^44^ In contrast, Vossen *et al.*^43^ found that heterozygous carriers of either Prothrombin G20210A or Factor V Leiden had a reduced risk of colorectal cancer. Using an MR Wald ratio analysis, we found very weak evidence that the Factor V Leiden allele was associated with a slightly reduced risk of colorectal cancer [OR 0.94 (95% CI 0.89–1.00), *P *=* *0.04, *FDR-P *= 0.59]. Asymptomatic carriers of Factor V Leiden have been shown to have accelerated formation of activated protein C.^61^ This enzyme has effects on endothelial barrier integrity and inflammation which appear to be independent of coagulation pathways.^62^ Therefore, the inverse association between Factor V Leiden and colorectal cancer risk may result from a biological interaction which is independent of thrombosis. Alternatively, the result could reflect confounding by population stratification. Prothrombin G20210A genotype data were only available for 12 of the 18 cancers; however, we did not find any associations between this variant and cancer as assessed by MR Wald ratios.

## Conclusions

We present a bidirectional MR analysis examining associations between genetic liability to VTE and 18 different cancers, using summary data from large GWAS meta-analyses. Our findings do not support the hypothesis that genetic liability to VTE is a cause of cancer. Genetic liability to VTE was associated with increased risk of pancreatic cancer and slightly reduced risk of oral cancer, but there was inadequate evidence to suggest a causal relationship. Further work is required to establish whether and how biological pathways involving ABO blood group contribute to epidemiological associations between VTE and pancreatic cancer. Additional mechanistic studies may elucidate causal relationships between active cancer and VTE, as well as the role of VTE in cancer progression.

## Ethics approval

This analysis used GWAS summary data only, and therefore no additional ethics approval was required. Approval was obtained by the original GWAS studies by their appropriate ethics review boards: see details in the source GWAS publications.

## Supplementary Material

dyad170_Supplementary_Data

## Data Availability

R scripts used for the analysis are available via GitHub [https://github.com/NaomiC-0/Mendelian-randomization-analysis-of-VTE-and-Cancer]. Harmonied summary data for all SNPs included in this analysis are available in the Supplementary data. Full summary statistics are publicly available via: the Open GWAS database [https://gwas.mrcieu.ac.uk] for ovarian cancer (accession number: ieu-a-1120) and prostate cancer (accession number: ieu-b-85); the European Bioinformatics Institute GWAS Catalogue [https://www.ebi.ac.uk/gwas] for endometrial cancer (accession number: GCST006464), lung cancer (accession number: GCST004748) and oesophageal cancer (accession number: GCST003739); and the Breast Cancer Association Consortium [https://bcac.ccge.medschl.cam.ac.uk] for breast cancer. PanScan and PanC4 individual-level data are available through dbGaP (https://www.ncbi.nlm.nih.gov.gap/, accession numbers phs000206.v5.p3 and phs000648.v1.p1, respectively). Application to the relevant GWAS consortium is required for full summary statistics for the remaining phenotypes.

## Disclaimer

Where authors are identified as personnel of the International Agency for Research on Cancer/World Health Organization, the authors alone are responsible for the views expressed in this article, and they do not necessarily represent the decisions, policy or views of the International Agency for Research on Cancer/World Health Organization. Where authors are identified as affiliated with the National Heart, Lung and Blood Institute or the National Institute of Health, the views expressed in this manuscript are those of the authors and do not necessarily represent the views of the National Heart, Lung and Blood Institute or the National Institute of Health.
