# Supplementary material for: Causal relationships between risk of venous thromboembolism and 18 cancers: a bidirectional Mendelian randomization analysis
Source: Int J Epidemiol. 2023 Dec 20;53(1):dyad170. doi: 10.1093/ije/dyad170 (PMC10859161; doi:10.1093/ije/dyad170)
Supplement: dyad170_Supplementary_Data [file dyad170_supplementary_data.zip › ije-2023-05-0578-File009.docx]

**Supplementary Methods**

### Data sources and Genetic Instruments

We obtained European-ancestry summary genetic data from genome-wide association study (GWAS) meta-analyses examining risk of venous thromboembolism (VTE) and 18 common cancers, respectively (**Table 1**). Summary genetic data included: the effect size of each single nucleotide polymorphism (SNP), expressed as a log-odds ratio; the standard-error of the effect size; *P*-value; sample size (including case-control ratio); and, where available, the effect allele frequency in the GWAS population. Case definitions and co-variate adjustment for each GWAS are shown in **Supplementary Table S1.**

To examine the association between genetic liability to VTE and each cancer, we extracted risk SNPs associated with VTE at *P*<5x10^-8^ from a VTE GWAS conducted by Thibord *et al*.[1] We clumped SNPs using a strict linkage disequilibrium (LD) threshold of r^2^<0.001 (10,000kb sliding window) to ensure independence. Clumping was performed with the ‘TwoSampleMR’ R package[2] using European-ancestry reference panels from the 1000 Genomes Project.[3] If exposure SNPs were absent from the reference panel, these were excluded from the analysis.

Summary statistics for the SNPs in our VTE IV were then extracted from each cancer risk GWAS (Table 1). If a VTE-risk SNP was not present in the GWAS summary statistics for a given cancer, an alternative SNP in high LD with the target SNP (r^2^ ≥0.8) was used as a proxy (if available). We harmonised exposure and outcome data to ensure that effect estimates corresponded to the same allele for each SNP across the VTE and cancer datasets. Coding-strand ambiguities for palindromic SNPs were resolved using effect allele frequencies if possible; palindromic SNPs with intermediate effect allele frequencies (0.42 – 0.58) were excluded.[2] Effect allele frequencies were not available for the oesophageal cancer and glioma GWAS. For oesophageal cancer we confirmed the coding-strand with study authors to facilitate data-harmonisation; for glioma all palindromic SNPs were excluded.

To perform the Mendelian randomisation analyses in the opposite direction (with genetic liability to cancer as an exposure and VTE as an outcome), we used the same process and thresholds described above to select independent GWAS-significant risk SNPs for each cancer from the relevant cancer GWAS, then looked up summary statistics for the cancer-risk SNPs in the VTE GWAS.

**References**

1. Thibord F, Klarin D, Brody JA *et al.* Cross-Ancestry Investigation of Venous Thromboembolism Genomic Predictors. *Circulation* 2022**; 146:** 1225-42

2. Hemani G, Zheng J, Elsworth B *et al.* The MR-Base platform supports systematic causal inference across the human phenome. Loos R (ed.). *eLife* 2018;**7**:e34408.

3. Auton A, Abecasis GR, Altshuler DM *et al.* A global reference for human genetic variation. *Nature* 2015;**526**:68–74.
